# Multidimensional repair of jujube pectic oligosaccharides on bone marrow hematopoietic failure

**DOI:** 10.3389/fnut.2026.1734800

**Published:** 2026-01-29

**Authors:** Hongxi Chen, BiYing Wang, XianZhen Li

**Affiliations:** 1 School of Bioengineering, Dalian Polytechnic University, Dalian, China; 2 College of Food Science and Engineering, Tarim University, Alar, Xinjiang, China

**Keywords:** anemia, cytokines, hematopoietic microenvironment, jujube pectic oligosaccharides, multi-target regulation

## Abstract

Jujube, a valued resource in traditional practices for both medicine and diet, has been historically recognized for its blood-nourishing properties. Nevertheless, the potential of its active constituent, Jujube Pectic Oligosaccharides (JOL), to ameliorate myelosuppressive anemia remains poorly understood. This research was therefore designed to elucidate the therapeutic efficacy and underlying mechanism of JOL using a murine model of cyclophosphamide-induced myelosuppression. Our findings demonstrate that JOL administration effectively restored multi-lineage peripheral blood cell counts, improved the structural integrity of the spleen and bone marrow, and modulated key hematopoietic factors. These critical factors comprised erythropoietin (EPO), Flt3 ligand (Flt3-L), thrombopoietin (TPO), and granulocyte colony-stimulating factor (G-CSF). Collectively, the results indicate that Jujube oligosaccharides could mitigate myelosuppressive anemia via synergistic multi-target effects, possibly by rehabilitating the damaged hematopoietic microenvironment and normalizing the cytokine network equilibrium. This investigation offers foundational experimental support for the development of JOL as a promising therapeutic candidate for myelosuppression.

## 1 Introduction

Anemia continues to be a critical global public health issue, with its persistently high prevalence posing ongoing risks to both human health and socioeconomic development. As reported by the World Health Organization, more than a billion individuals globally live with anemia (1). The condition is pathologically defined by a reduction in red blood cell count or hemoglobin concentration to below-normal levels (2). This hematologic disturbance directly compromises the blood's oxygen-carrying capacity, inducing systemic chronic hypoxia that presents clinically through symptoms including persistent fatigue, general weakness, dizziness, and palpitations (3). Such manifestations not only considerably reduce daily functioning and well-being but also impose a significant healthcare and socioeconomic burden.

Among the various forms of anemia, hematopoietic hypoplastic anemia presents particular clinical difficulty, as it stems from a fundamental failure in bone marrow hematopoiesis and is frequently associated with unfavorable outcomes. The causes of this anemia subtype are multifactorial, ranging from exposure to chemical agents (including anticancer chemotherapy drugs), ionizing radiation, and environmental toxins, to specific viral infections and autoimmune disorders (4, 5). The central pathophysiological process entails the exhaustion of the hematopoietic stem and progenitor cell compartment, suppression of their proliferative capacity, or disruption of normal differentiation pathways, which collectively cause a failure in the downstream production of mature blood cells (6).

The current clinical management of hematopoietic hypoplastic anemia relies on several cornerstone therapies. Transfusion support, primarily through blood components, offers immediate correction of severe hematologic deficits (7); however, its benefits are short-lived, and prolonged dependence carries risks of complications including transfusion-transmitted infections, alloimmunization, and iron overload. Another mainstay treatment involves the administration of hematopoietic growth factors—for instance, recombinant human erythropoietin and granulocyte colony-stimulating factor—which act by selectively stimulating the proliferation and differentiation of specific hematopoietic lineages (8).

However, their clinical utility is often constrained by high cost, diminishing efficacy over time (e.g., EPO resistance), and side effects such as bone pain, hypertension, and thrombosis (9, 10). Thirdly, for immune-mediated aplastic anemia, intensive immunosuppressive therapy is the cornerstone, yet the consequent risk of systemic immunodeficiency cannot be ignored. Furthermore, etiological treatments for nutritional anemias (e.g., iron, vitamin B12, or folate supplementation), while effective, are not applicable to hematopoietic suppression caused by non-nutritional factors (11). Consequently, developing novel therapeutic agents or adjuvant strategies that are highly effective, safe, and economical represents a pressing unmet clinical need in this field.

Given these limitations, natural products have garnered research interest as complementary agents for managing hematological diseases, due to their wide availability, multifaceted biological activities, and favorable safety profiles (12). Among them, jujube (Ziziphus jujuba Mill.) holds a significant position. It is a prime example of a “medicinal and edible” substance in Traditional Chinese Medicine, with a documented history of application in fortifying the middle *Jiao* and boosting Qi (13), as well as in blood nourishment and mental sedation. Modern pharmacological research has gradually unveiled its scientific basis: jujube is rich in various bioactive compounds including polysaccharides, saponins, flavonoids, and cyclic nucleotides. Among these, jujube polysaccharides have been extensively demonstrated to possess multiple pharmacological activities, such as immunomodulation, antioxidation, antitumor effects, as well as promoting hematopoiesis and anti-fatigue properties (14, 15). However, polysaccharides generally face challenges like large molecular weight, complex structure, and low absorption rate *in vivo*, which to some extent limit their bioavailability and potential for clinical application.

In recent years, oligosaccharides, as degradation products of polysaccharides, have become a new focus in natural product research due to their smaller molecular weight, good water solubility, ease of absorption and utilization by the body, and demonstration of various remarkable biological activities such as prebiotic, immunomodulatory, and antioxidant effects (16). Jujube oligosaccharides (JOL) are low-degree polymerization sugar chain fragments obtained through the controlled degradation (physical, chemical, or enzymatic) of jujube polysaccharides (16, 17). Preliminary research suggests that JOL can not only retain the core biological activities of jujube polysaccharides but might even exhibit enhanced efficacy due to their superior biological properties, showing significant potential particularly in regulating gut microbiota and enhancing immune function. However, whether JOL can directly act on the impaired hematopoietic system and exert a definitive therapeutic effect against hematopoietic hypoplastic anemia—and through what mechanism—remains unclear due to a lack of systematic evidence.

Based on the aforementioned research status and scientific hypothesis, this study aims to systematically evaluate the therapeutic effect of Jujube oligosaccharides (JOL) on a robust hematopoietic suppression mouse model and preliminarily explore its potential pathways of action. The significance of this research lies in being the first to focus on JOL, a small-molecular-weight active component derived from jujube, and to deeply investigate its direct efficacy in treating hematopoietic hypoplastic anemia. The findings are expected to provide key evidence for elucidating the hematopoietic protective and reparative mechanisms of JOL, and to lay a solid theoretical and experimental foundation for developing safe, efficient, novel anemia treatment resources or adjuvant therapeutic strategies based on natural oligosaccharides.

## 2 Materials and methods

### 2.1 Mouse studies

Forty 3-week-old male Kunming mice of SPF grade were purchased from Liaoning Changsheng Biotechnology Co., Ltd. (Production License No.: SCXK (Liao) 2025-0001). All mice were acclimatized for 1 week under standard laboratory conditions with ambient temperature controlled at 20 °C−25 °C, relative humidity at 40%−50%, and a 12-h light/dark cycle. Mice had free access to standard rodent diet and sterile drinking water. All experimental procedures were approved by the Institutional Animal Ethics and Use Committee of Qingdao Harwars Biology Group Ltd. (Approval No.: AUP-QY-C-S-2025-007).

The positive control drug, Ass-hide Gelatin (Ejiao), was purchased from Beijing Tongrentang Co., Ltd. The jujube pectic oligosaccharides (JOL) used in this study were prepared in our laboratory according to the following protocol: dried jujube fruits (Ziziphus jujuba Mill. cv. “Jinsixiaozao”) from Laoling City, Shandong Province, China, were used as the raw material. The fruits were first extracted with hot water (80 °C) for 1 h. This extraction was repeated twice on the residue. The combined aqueous extracts were concentrated and precipitated with three volumes of ethanol. After storage at 4 °C overnight, the precipitate was collected by centrifugation, freeze-dried, and obtained as crude jujube polysaccharide. This crude polysaccharide was then subjected to enzymatic hydrolysis to produce oligosaccharides. The hydrolysis was performed using a laboratory-prepared pectinase (enzyme activity = 300 U/ml) at 45 °C for 24 h. The reaction was terminated by heating. The hydrolysate was further purified through a series of steps, including ethanol precipitation, deproteinization using the Sevag method, and fractionation with a 1 kDa molecular weight cut-off ultrafiltration membrane to obtain the target pectic oligosaccharide fraction. The final product was characterized by matrix-assisted laser desorption/ionization time-of-flight mass spectrometry (MALDI-TOF-MS), which confirmed that the molecular weights of the JOL were primarily distributed within the range of 666 to 893 Da.

The dose of JOL (100 mg/kg) was selected based on preliminary experiments and literature reports on the bioactivity of similar oligosaccharides. Ass-hide Gelatin (Ejiao), a well-established hematinic agent in Traditional Chinese Medicine for treating blood deficiency syndromes, was chosen as a positive control at its commonly used effective dose (100 mg/kg) to provide a clinically relevant benchmark for efficacy comparison. Prior to intragastric administration, Ass-hide Gelatin, JOL, and jujube polysaccharides were dissolved in physiological saline to achieve solutions of the required concentrations.

Following the acclimatization phase, the mice were weight-stratified and randomly allocated into four groups (*n* = 8 per group): a Normal Control group and three groups designated for model establishment. The modeling procedure was conducted following a previously reported method (18). Briefly, mice in the three model groups were administered daily intraperitoneal injections of a cyclophosphamide solution (100 mg/kg) over four consecutive days to induce myelosuppressive anemia. Concurrently, mice in the Normal Control group received intraperitoneal injections of an equivalent volume of physiological saline.

Model validation was performed 24 h after the final cyclophosphamide injection. Tail vein blood samples were collected and analyzed using an automated hematology analyzer. A successful model was confirmed based on established criteria (19), characterized by a significant reduction in white blood cell count, hemoglobin concentration, and platelet count compared to the Normal Control group.

Mice that met the modeling criteria were subsequently re-randomized into three experimental groups: a Model group (receiving no treatment), an AE group (Ass-hide Extract, 100 mg/kg), and a JOL group (Jujube Oligosaccharide, 100 mg/kg).

The therapeutic intervention was initiated the day after model confirmation. Daily at 10:00 a.m., the AE and JOL groups received their respective drug solutions via oral gavage (10 ml/kg). In parallel, the Normal Control and Model groups were administered an equal volume of physiological saline. This treatment regimen continued once daily for seven consecutive days.

Upon completion of the 7-day treatment period, all mice were fasted for 12 h with free access to water. Subsequently, the animals were euthanized by cervical dislocation, and the required biological samples were collected for further analysis.

**Peripheral blood:** used for routine blood analysis.

**Bone marrow cell suspension:** bone marrow cells were harvested by flushing the medullary cavities of both femurs and tibias with a sterile saline solution to obtain a single-cell suspension.

**Organ collection and analysis:** the thymus and spleen were aseptically excised and weighed for the calculation of the organ index (organ mass/body mass, mg/g). A portion of the spleen tissue was immediately fixed in 4% paraformaldehyde for subsequent processing, which included paraffin embedding, sectioning, and hematoxylin and eosin (H&E) staining to enable histopathological evaluation.

**Bone marrow histology:** one tibia from each mouse was collected and fixed. Following a standard decalcification process, the bone specimen was embedded in paraffin, sectioned, and stained with H&E to allow for detailed examination of the bone marrow histoarchitecture and cellular morphology.

The study encompassed a comprehensive set of evaluations, including: complete blood count analysis, quantification of key hematopoietic factor (e.g., EPO, GM-CSF) expression levels within the bone marrow, histopathological assessment of tibial bone marrow sections, calculation of thymus and spleen indices, and microscopic examination of pathological changes in spleen tissue sections.

### 2.2 Hematological analysis

Tail vein blood samples (20 μl) were collected into EDTA-K2 anticoagulant tubes and subjected to a comprehensive hematological profile analysis using an automated hematology analyzer. The assessed parameters encompassed:

**White blood cell series:** including white blood cell count (WBC), lymphocyte count (LYM), granulocyte count (GR), and mid-range cell count (MID).

**Red blood cell series:** comprising red blood cell count (RBC), hemoglobin concentration (HGB), mean corpuscular volume (MCV), red cell distribution width coefficient of variation (RDW-CV), hematocrit (HCT), and mean corpuscular hemoglobin concentration (MCHC).

**Platelet series:** covering platelet count (PLT), platelet large cell ratio (P-LCR), plateletcrit (PCT), and platelet distribution width (PDW).

### 2.3 Spleen and thymus indices

Upon completion of the experimental protocol, the final body weight of each mouse was recorded. After euthanasia, the spleen and thymus were meticulously dissected and their wet weights were immediately obtained. The organ indices, reflecting relative organ mass, were subsequently calculated as the ratio of organ weight (mg) to body weight (g).

Spleen Index (%) = (Spleen Weight/Body Weight) × 100%

Thymus Index (%) = (Thymus Weight/Body Weight) × 100%.

### 2.4 Histological examination of spleen tissue

Spleens were harvested post-sacrifice and fixed in 4% paraformaldehyde. The fixed tissues were then processed through standard histological procedures for paraffin embedding and sectioning. The obtained sections were subjected to the following staining protocols for detailed analysis:

Hematoxylin and Eosin (H&E) Staining: to evaluate the general histological architecture of the spleen and identify the presence of extramedullary hematopoiesis.

Prussian blue staining: to detect and localize iron deposits (hemosiderin) within the splenic tissue.

Reticular fiber staining: utilizing a silver impregnation method to delineate the supporting reticular fiber network of the spleen.

Following staining, all sections underwent a series of steps including dehydration, clearing, and mounting with a neutral balsam. The prepared slides were then examined and imaged using a standard light microscope for subsequent analysis.

### 2.5 Bone marrow sectioning and analysis of hematopoietic factors

For histological examination, femoral bones were dissected and underwent fixation in 4% paraformaldehyde over a 48-h period. Subsequently, the fixed bone samples were decalcified, dehydrated through a graded ethanol series, and embedded in paraffin wax. Sections were cut at a thickness of 4 μm and stained with hematoxylin and eosin (H&E) to facilitate microscopic evaluation of histopathological alterations within the bone marrow microenvironment.

For the quantification of hematopoietic factors, bone marrow cells were harvested from both femurs and tibias by perfusing the medullary cavities with ice-cold phosphate-buffered saline (PBS). The resulting cell suspensions were centrifuged at 3,000 rpm for 10 min at 4 °C. The acquired supernatant, designated as the bone marrow flush fluid, was carefully aliquoted and stored at −80 °C to preserve analyte integrity until further analysis.

The levels of key hematopoietic factors in the bone marrow flush fluid were determined using commercially available Enzyme-Linked Immunosorbent Assay (ELISA) kits, in strict adherence to the protocols provided by the manufacturer. The panel of analytes measured included: Erythropoietin (EPO), Thrombopoietin (TPO), Granulocyte Colony-Stimulating Factor (G-CSF), Granulocyte-Macrophage Colony-Stimulating Factor (GM-CSF), Fms-related tyrosine kinase 3 ligand (Flt3-L), Interferon-gamma (IFN-γ), and Tumor Necrosis Factor-alpha (TNF-α).

### 2.6 Statistical analysis

All quantitative data are presented as the mean ± standard deviation (SD). Statistical significance among multiple groups was determined by one-way analysis of variance (ANOVA) followed by Tukey's *post hoc* test for multiple comparisons, using GraphPad Prism software (version 10.6.1, GraphPad Software, USA). A *p*-value of less than 0.05 (*p* < 0.05) was considered statistically significant. The specific statistical tests are indicated in the respective figure legends.

## 3 Results

### 3.1 Evaluation of systemic and immuno-organ parameters: body weight, thymus, and spleen indices

To assess the systemic impact of cyclophosphamide-induced myelosuppression and the efficacy of therapeutic interventions, we tracked the longitudinal changes in body weight and computed the indices of the thymus and spleen as key indicators of immune organ status.

**Figure 1:**
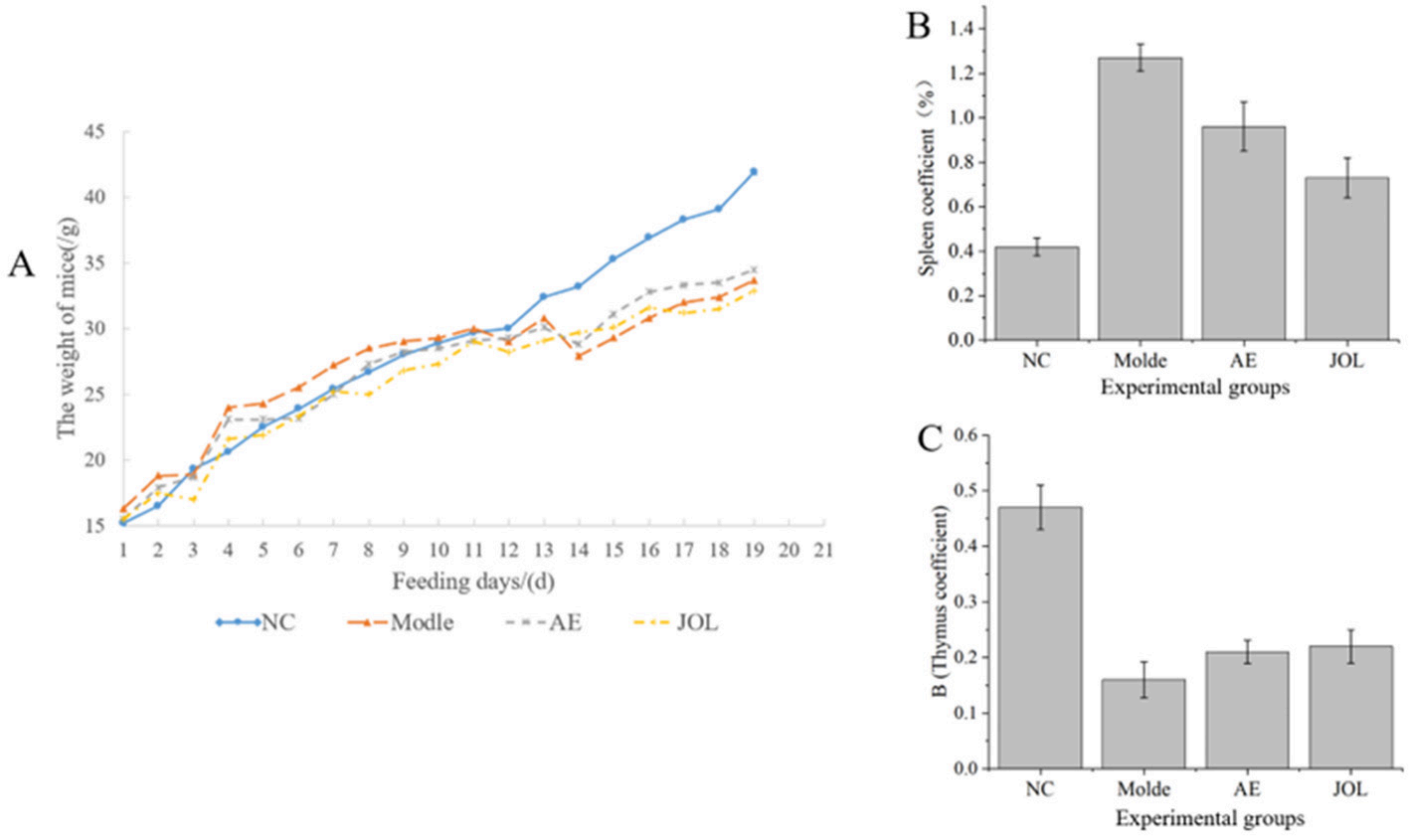
**(A)** Body weight changes, **(B)** spleen index, and **(C)** thymus index in mice from different groups over the experimental timeline.

Body weight dynamics: the progression of body weight across experimental groups is depicted in Figure 1A. Mice in the Normal Control (NC) group maintained a consistent growth trajectory, gaining weight steadily throughout the study. In stark contrast, during the modeling phase (Days 8–12), animals in the Model group displayed a markedly attenuated rate of weight gain, which nearly ceased toward the end of this phase. During the subsequent treatment period (Days 13–19), the Model group experienced a decline in body mass, reflecting a worsening systemic condition. In contrast, all therapeutic intervention groups (AE, JOL, JPS) reversed this trend and showed restorative weight gain. Notably, the Jujube Oligosaccharide (JOL) group displayed the most robust recovery, implying a superior capacity of JOL to ameliorate the overall physiological decline in myelosuppressed mice.

**Spleen index:** the spleen index serves as a critical parameter for evaluating splenic pathophysiology. In myelosuppressive anemia, the failure of bone marrow hematopoiesis often triggers compensatory extramedullary hematopoiesis within the spleen, leading to organ enlargement (20). Furthermore, anemia-induced reductions in red blood cell lifespan and their subsequent sequestration can exacerbate splenic congestion and hypertrophy (21). Consistent with these pathophysiological events, our data (Figure 1B) revealed a significant increase in the spleen index across all model groups compared to the NC group, validating successful model establishment. Following 1 week of treatment, both the Ass-hide Gelatin (AE) and Jujube Oligosaccharide (JOL) groups exhibited a significant reduction in spleen index compared to the untreated Model group. The spleen index in the JOL group, in particular, was restored to a level most closely approximating that of the NC group. These findings suggest that JOL and AE treatments may facilitate the normalization of splenic size, potentially by alleviating the compensatory burden on the spleen or directly improving the anemic condition, possibly through mechanisms involving bone marrow microenvironment repair or reduction of pathological erythrocytes, as will be explored in subsequent histological and hematological analyses.

**Thymus index:** while not directly involved in hematopoiesis, the thymus index provides an indirect measure of systemic immune competence (22). As illustrated in Figure 1C, a significant reduction in the thymus index was observed in the Model group relative to the NC group, demonstrating that cyclophosphamide-induced damage extends beyond the bone marrow to impair this primary lymphoid organ. Post-therapeutic intervention, all treatment groups showed a modest upward trend in thymus index. This suggests a potential beneficial role of the drug treatments in supporting the recovery of immune function in model mice, the full implications of which warrant further investigation with more definitive immunological assays.

### 3.2 Restoration of peripheral blood homeostasis by jujube oligosaccharides in myelosuppressed mice

To determine the therapeutic efficacy of Jujube Oligosaccharides (JOL) against cyclophosphamide-induced myelosuppressive anemia, a comprehensive analysis of post-treatment peripheral blood parameters was conducted. The evaluated parameters are detailed across three major lineages: white blood cells, red blood cells, and platelets.

#### 3.2.1 Recovery of the leukocyte lineage

**Figure 2:**
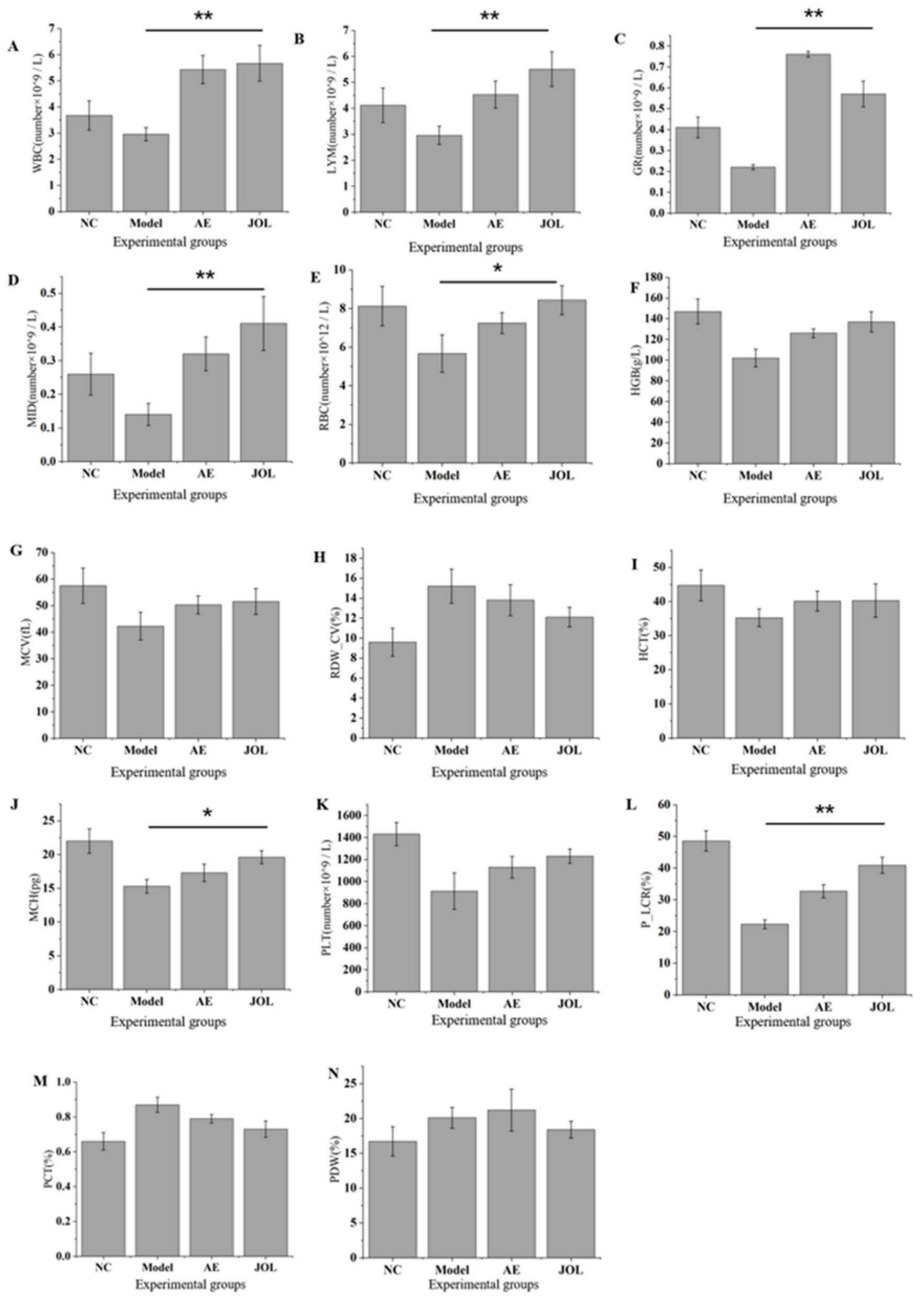
Peripheral blood parameters in mice: **(A)** WBC (white blood cell count), **(B)** LYM (lymphocyte count), **(C)** GR (granulocyte count), **(D)** MID (Mid-range cell count), **(E)** RBC (red blood cell count), **(F)** HGB (hemoglobin concentration), **(G)** MCV (mean corpuscular volume), **(H)** RDW-CV (red cell distribution width), **(I)** HCT (hematocrit), **(J)** MCH (mean corpuscular hemoglobin), **(K)** PLT (platelet count), **(L)** P-LCR (platelet-large cell ratio), **(M)** PCT (plateletcrit), and **(N)** PDW (platelet distribution width). * indicates *p* < 0.05. ** indicates *p* < 0.01.

Data presented in Figures 2A–D reveal that cyclophosphamide administration caused a severe suppression of hematopoiesis, as evidenced by significantly reduced counts of white blood cells (WBC), lymphocytes (LYM), granulocytes (GR), and mid-range cells (MID) in the Model group compared to the Normal Control (NC) group. Following the intervention, all treatment groups displayed a measurable recovery in these parameters. The JOL group, in particular, demonstrated the most prominent upregulation in total WBC and LYM counts (Figures 2A, B). The rapid rebound of leukocytes is a recognized early indicator of hematopoietic stimulation. Specifically, the restoration of granulocytes implies revived granulopoiesis; the recovery of lymphocytes points to the reconstitution of adaptive immunity and a bolstered antigen response (23); and the increase in MID cells (encompassing monocytes, etc.) signals the replenishment of macrophage precursors (24), which are crucial for clearing cellular debris and facilitating tissue repair. Collectively, the elevation in total WBC count provides direct evidence for a revitalized bone marrow hematopoietic microenvironment.

#### 3.2.2 Amelioration of erythrocyte parameters

Key erythrocytic indices are central to evaluating the severity of anemia. As illustrated in Figures 2E–J, mice in the Model group exhibited a sharp decline in red blood cell count (RBC), hemoglobin concentration (HGB), and hematocrit (HCT), confirming a state of severe anemia. Administration of JOL resulted in the most substantial improvement across these three core parameters (Figures 2E–G). Notably, the Mean Corpuscular Volume (MCV) and Mean Corpuscular Hemoglobin Concentration (MCHC) remained largely unaltered across the model groups compared to the NC group (Figures 2H, I), characterizing the anemia as predominantly normocytic and normochromic. Furthermore, the elevated Red cell distribution width-coefficient of variation (RDW-CV) in the Model group indicated heightened heterogeneity in red cell size (anisocytosis), a finding consistent with the cytotoxic action of cyclophosphamide. This also correlates with the observed splenomegaly, as the spleen's workload in clearing these abnormal erythrocytes is increased. The subsequent reduction in RDW-CV following JOL treatment underscores its therapeutic benefit in promoting stable and uniform erythropoiesis.

#### 3.2.3 Kinetics of platelet-lineage regeneration

Tracking platelet-related parameters offers valuable insights into the dynamics of bone marrow injury and subsequent repair. According to the results in Figures 2K–N, the Model group showed a pronounced reduction in Platelet count (PLT) and Plateletcrit (PCT), a hallmark of compromised megakaryopoiesis. Although all treatment groups showed only modest increases in PLT and PCT by the end of the study (Figures 2K, L), a critical positive shift was observed in other indices. A notable decrease in Platelet distribution width (PDW) coupled with a concurrent rise in the Platelet-large cell ratio (P-LCR) (Figures 2M, N) was detected. This specific pattern is a robust indicator of bone marrow recovery, signifying that megakaryocytes have resumed production and are releasing a fresh cohort of larger, more reactive platelets into the periphery (25). Thus, despite incomplete quantitative restoration within the experimental timeframe, this kinetic profile clearly signifies the reactivation of thrombopoietic function in the bone marrow.

### 3.3 Histopathological analysis

Histopathological analysis served as a cornerstone for validating the successful induction of myelosuppressive anemia and for elucidating the underlying pathological mechanisms (26). A multi-faceted staining approach was employed: bone marrow sections were assessed via Hematoxylin and Eosin (H&E) staining, while spleen tissues underwent a comprehensive evaluation using H&E, Prussian blue (Perls' stain) for iron detection, and MASSON trichrome staining for connective tissue.

#### 3.3.1 Alterations in bone marrow histoarchitecture

**Figure 3:**
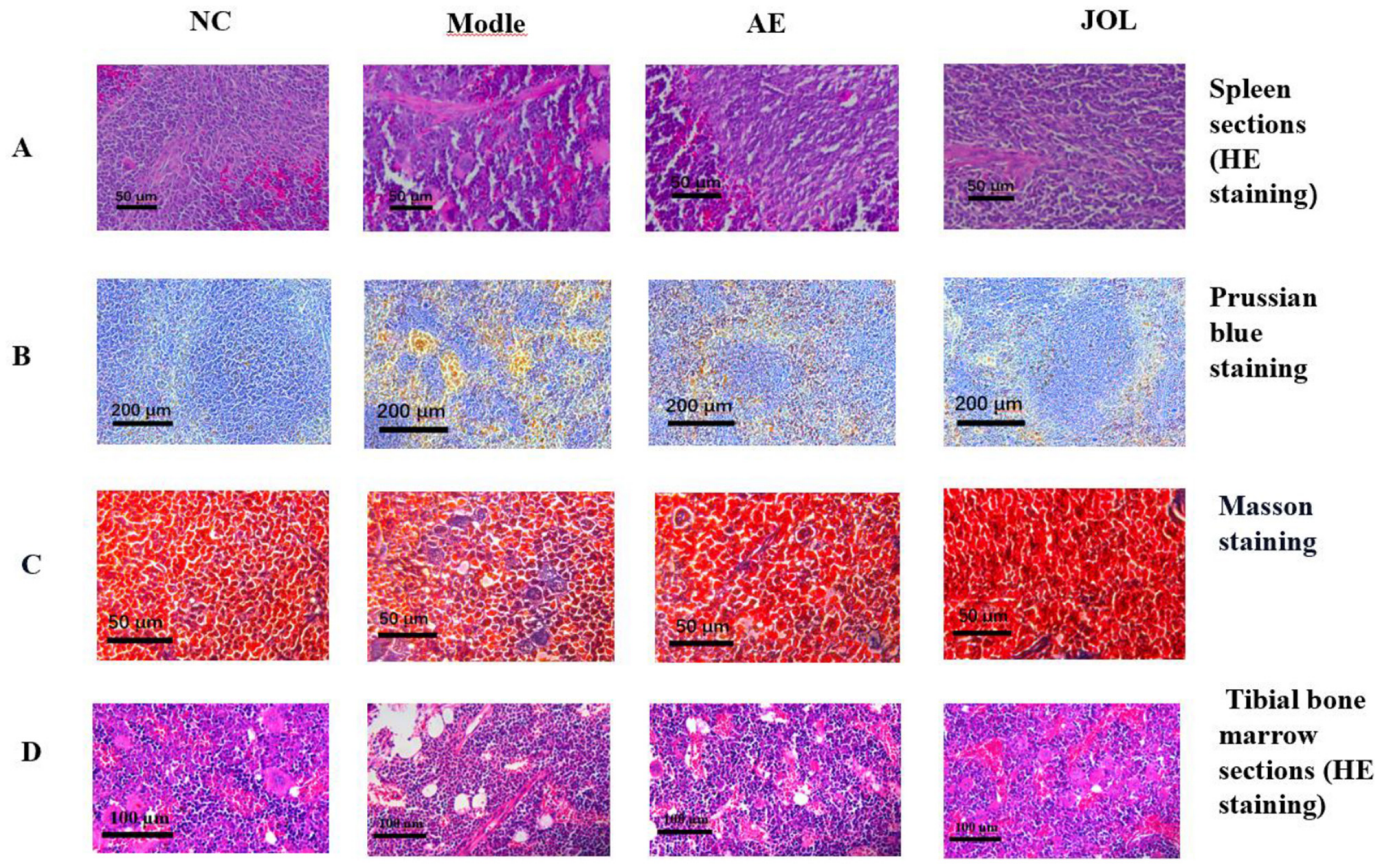
Histopathological sections of mouse spleen and bone marrow. **(A)** Spleen (H&E staining), **(B)** spleen (Prussian Blue staining for iron), **(C)** spleen (Masson's trichrome staining for collagen), and **(D)** bone marrow (H&E staining).

The bone marrow, being the primary site of hematopoiesis, offers a direct morphological reflection of its functional state (27). Tibial bone marrow sections from the Model group (Figure 3D) displayed severe hypoplasia relative to the Normal Control (NC) group. This was morphologically defined by a substantial depletion of nucleated hematopoietic cells, a concomitant rise in adipose tissue, and the conspicuous scarcity of erythroblastic islands. This “hypocellular marrow” phenotype provides definitive structural evidence for the cyclophosphamide-induced failure of bone marrow function, constituting the fundamental pathology behind the observed anemia and thrombocytopenia. Post-treatment analysis revealed that the Jujube Oligosaccharide (JOL) group exhibited the most substantial architectural recovery, evidenced by a repopulated hematopoietic cell compartment, diminished adipocyte vacuolization, and a restoration of densely packed hematopoietic tissue. The degree of histological improvement in the JOL group surpassed that observed in other treatment cohorts.

#### 3.3.2 Splenic histopathology: insights into extramedullary hematopoiesis and metabolism

Histological changes in the spleen provide critical insights into compensatory extramedullary hematopoiesis and systemic iron metabolism (28).

- **H&E staining:** analysis of H&E-stained sections (Figure 3A) revealed a marked expansion of the splenic red pulp in the Model group. This is a characteristic pathological adaptation, indicating the initiation of extramedullary hematopoiesis by the spleen to counterbalance the loss of bone marrow function, thereby attempting to mitigate the systemic anemic condition through increased erythrocyte production.
- **Prussian blue iron staining:** spleens from the Model group (Figure 3B) displayed abundant blue-stained hemosiderin granules upon Perls' staining. This finding provides clear morphological evidence of enhanced aberrant erythrocyte destruction in the anemic state, leading to dysregulated iron metabolism and consequent iron overload within the spleen. It is noteworthy that JOL intervention markedly attenuated this pathological iron deposition, implying its role in reducing red blood cell destruction and facilitating the restoration of iron homeostasis.
- **MASSON trichrome staining:** this technique was utilized to evaluate collagen fiber deposition, an indicator of tissue remodeling and fibrosis (29). Contrary to potential expectations, spleens from the Model group exhibited conspicuous proliferation of blue-stained collagen fibers (Figure 3C). This indicates that the acute stress of cyclophosphamide-induced myelosuppression triggers not only compensatory hematopoiesis but also pathological tissue remodeling and incipient fibrosis in the spleen, an organ burdened with immense clearance and hematopoietic loads. This finding offers an additional, crucial explanation for the observed splenomegaly beyond mere extramedullary hematopoiesis. Following therapeutic intervention, JOL-treated mice demonstrated a significant reduction in collagen fiber deposition, with splenic morphology closely resembling that of the NC group. This result strongly suggests that Jujube Oligosaccharides facilitate not only hematopoietic restoration but also directly or indirectly attenuate splenic fibrogenesis, thereby enabling a more holistic repair of splenic structure and function.

### 3.4 Changes in bone marrow hematopoietic factor levels

Quantifying the levels of bone marrow hematopoietic factors provides a dynamic and sensitive readout of the systemic hematopoietic status, injury mechanisms, and inherent compensatory capacity (30). To this end, we quantified a panel of seven pivotal cytokines in the bone marrow flush fluid. The panel comprised five stimulatory factors—Erythropoietin (EPO), Thrombopoietin (TPO), Granulocyte Colony-Stimulating Factor (G-CSF), Granulocyte-Macrophage Colony-Stimulating Factor (GM-CSF), and Fms-related tyrosine kinase 3 ligand (Flt3-L)—alongside two inhibitory inflammatory cytokines, Interferon-gamma (IFN-γ) and Tumor Necrosis Factor-alpha (TNF-α).

#### 3.4.1 Upregulation of stimulatory hematopoietic growth factors

**Figure 4:**
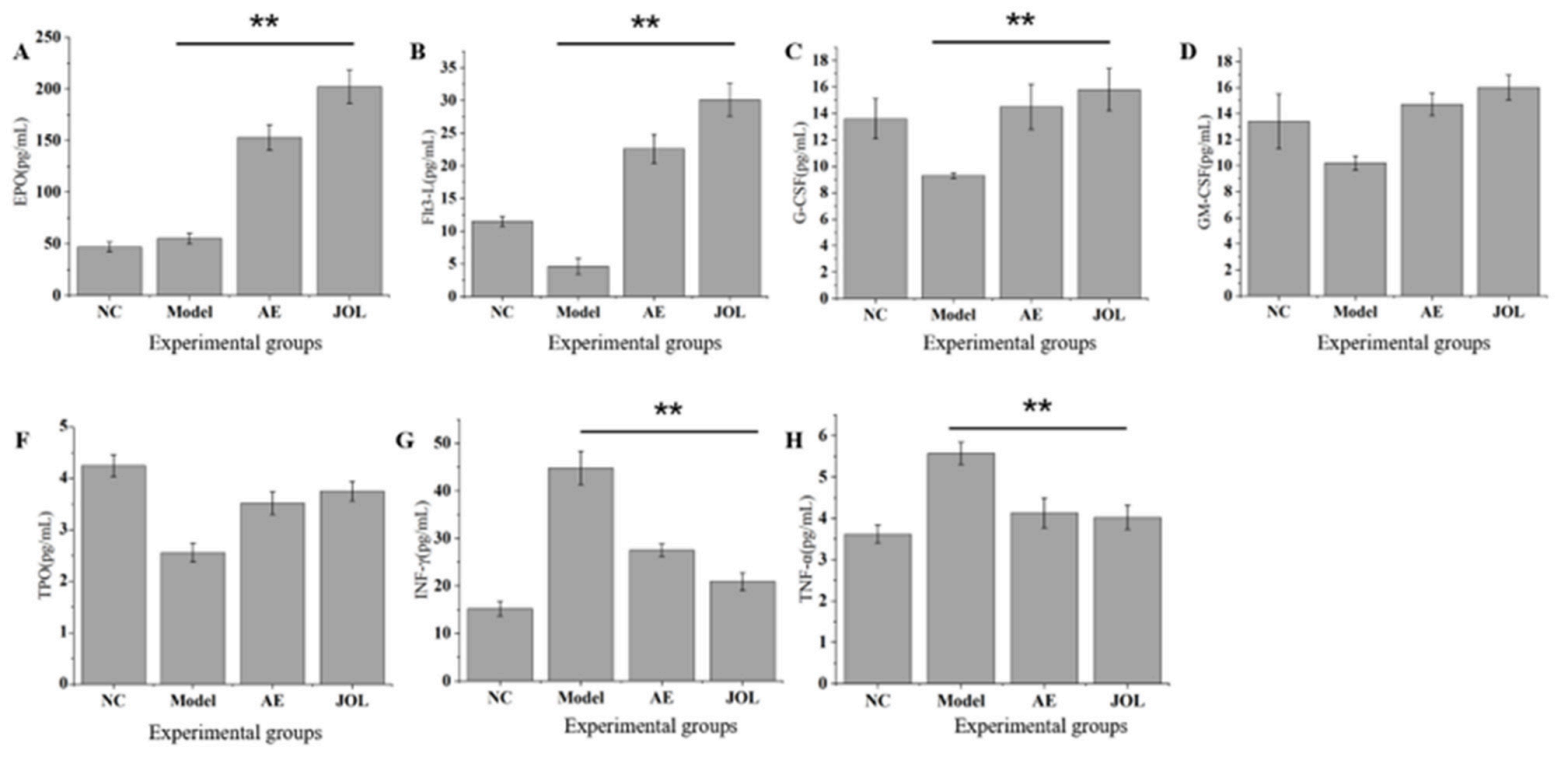
Levels of hematopoietic factors in mouse bone marrow: **(A)** EPO (Erythropoietin), **(B)** Flt3-L (Fms-related tyrosine kinase 3 ligand), **(C)** G-CSF (granulocyte colony-stimulating factor), **(D)** GM-CSF (granulocyte macrophage colony-stimulating factor), **(E)** TPO (Thrombopoietin), **(F)** IFN-γ(Interferon-gamma), **(G)** TNF-α (tumor necrosis factor-alpha). * indicates *p* < 0.05. ** indicates *p* < 0.01.

Analysis of bone marrow flush fluid revealed that treatment with Jujube Oligosaccharides (JOL) prompted an upward trend in the concentrations of several key hematopoietic growth factors relative to the Model group (Figures 4A–E). The most pronounced elevations were observed for EPO and Flt3-L (Figures 4A, B). The marked increase in EPO constitutes a robust physiological countermeasure to severe anemia, aimed at maximally stimulating the proliferation and differentiation of erythroid precursors. Conversely, the significant upregulation of Flt3-L is typically indicative of substantial injury to the hematopoietic stem and progenitor cell (HSPC) compartment, demanding potent signaling to drive its self-renewal and repopulation. This observation further elucidates the intrinsic difficulty in spontaneous repair and the poor baseline recovery capacity observed in this myelosuppressive anemia model.

In contrast to EPO and Flt3-L, the increase in TPO was marginal and statistically non-significant (Figure 4C). This attenuated response may originate from the profound damage cyclophosphamide inflicts upon megakaryocytic precursors, potentially disrupting the physiological feedback mechanism wherein platelet mass regulates TPO production. A moderate recovery of G-CSF was noted in the JOL group (Figure 4D), reflecting a compensatory response to neutropenia. Meanwhile, a more substantial increasing trend was observed for GM-CSF (Figure 4E), a cytokine with broader target cell specificity than G-CSF (31). The body may therefore upregulate GM-CSF to synergistically drive the regeneration of the entire myeloid lineage, encompassing granulocytes and monocytes/macrophages, potentially compensating for the relatively subdued G-CSF response.

#### 3.4.2 Attenuation of inhibitory inflammatory cytokines

In terms of negative regulators, the model group displayed a significant elevation in the bone marrow concentrations of the pro-inflammatory cytokines IFN-γ and TNF-α (Figures 4F, G), which are known to directly suppress hematopoietic stem cell proliferation and disrupt the integrity of the marrow microenvironment (32). Following JOL intervention, the levels of these inflammatory mediators were markedly suppressed. This indicates that the therapeutic efficacy of JOL involves mitigating the aberrant immune activation and inflammatory state induced by cyclophosphamide, thereby fostering a more favorable milieu for the recovery of hematopoietic function.

These data indicate that JOL modulates the bone marrow cytokine milieu in association with functional recovery. While this correlation supports a potential mechanistic role, direct causal evidence remains to be established in future interventional studies.

## 4 Discussion

This study provides systematic evidence for the therapeutic potential of jujube pectic oligosaccharides (JOL) in a murine model of cyclophosphamide-induced myelosuppressive anemia. Our experimental data demonstrate that JOL intervention led to the following key observations: (1) restoration of body weight loss and alleviation of splenomegaly; (2) significant multi-lineage recovery in peripheral blood counts, including white blood cells, red blood cells, and platelets; (3) repair of histopathological damage in both bone marrow and spleen tissues; and (4) modulation of the bone marrow cytokine milieu, characterized by an upregulation of key hematopoietic growth factors (EPO, Flt3-L) and a downregulation of pro-inflammatory cytokines (IFN-γ, TNF-α). Under the experimental conditions employed, the overall restorative efficacy of JOL was notable.

### 4.1 Orchestration of multi-lineage hematopoietic recovery

The most direct testament to JOL's efficacy is the pronounced recovery observed across peripheral blood lineages. The rapid resurgence of leukocytes, particularly lymphocytes, points to a potent stimulation of both the myeloid and lymphoid arms of hematopoiesis, likely heralding the re-establishment of robust immune competence (33). Critically, JOL exerted the most potent restorative effect on fundamental erythrocytic parameters—RBC, HGB, and HCT—concurrently normalizing the red cell distribution width (RDW-CV). This combination of findings indicates that JOL not only boosts the quantitative output of erythropoiesis but also enhances the qualitative uniformity and stability of the erythrocytes produced. Although full restitution of platelet counts may necessitate a longer treatment window, the definitive shift in platelet indices—specifically, a decreased platelet distribution width (PDW) and an elevated platelet-large cell ratio (P-LCR)—provides compelling evidence of reactivated megakaryopoiesis and the subsequent release of nascent, functionally robust platelets into the circulation.

### 4.2 Structural and functional reconstitution of the hematopoietic niche

Histopathological analyses furnish a morphological foundation for JOL's actions. The reversal of the cyclophosphamide-induced “hypocellular marrow” phenotype, evidenced by repopulated hematopoietic cells and diminished adipocyte infiltration in JOL-treated mice, constitutes direct proof of its capacity to repair the structural integrity of the hematopoietic inductive microenvironment. In parallel, splenic histology revealed that JOL mitigated the expansion of the red pulp, a hallmark of compensatory extramedullary hematopoiesis. Complementing this (34), Prussian blue staining demonstrated a significant clearance of pathological hemosiderin deposits. Together, these findings suggest that JOL restores the bone marrow's intrinsic hematopoietic capacity, thereby alleviating the spleen's compensatory burden, correcting iron dyshomeostasis from aberrant erythrocyte destruction, and guiding hematopoiesis back to its primary medullary site.

### 4.3 Harmonized regulation of the hematopoietic cytokine landscape

At a molecular level, the cytokine profile offers insights into JOL's mode of action. JOL promoted the expression of several pivotal hematopoietic growth factors. The marked elevation in EPO signifies a potent physiological countermeasure to anemia (35), which may be amplified by JOL. The substantial upregulation of Flt3-L strongly intimates that JOL targets the most primitive hematopoietic stem and progenitor cell (HSPC) compartment, facilitating its maintenance and expansion—a prerequisite for the sustained recovery of all blood lineages (36). The distinct modulation of factors like GM-CSF and G-CSF further suggests a sophisticated, coordinated regulation of myelopoiesis under JOL influence. The significant suppression of IFN-γ and TNF-α is particularly noteworthy. These pro-inflammatory cytokines are known to directly induce apoptosis of hematopoietic stem cells, impair the supportive function of bone marrow mesenchymal stromal cells (37, 38), and disrupt the niche integrity. Therefore, the anti-inflammatory effect of JOL likely creates a more permissive microenvironment for hematopoiesis to occur, synergizing with the upregulated growth factors.

**Figure 5:**
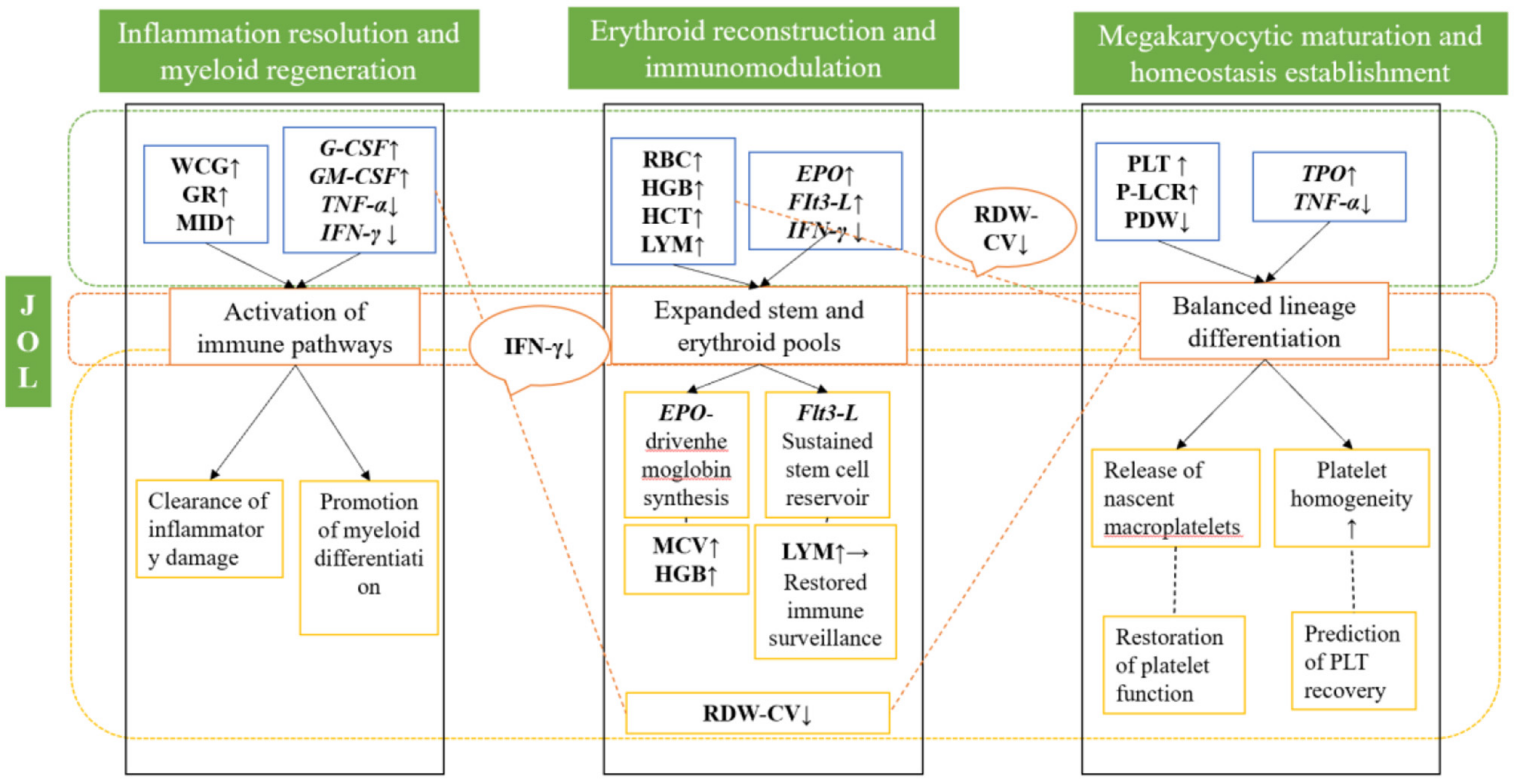
A hypothetical three-phase model illustrating the potential therapeutic mechanism of JOL in myelosuppressive anemia. This schematic model is proposed based on the correlative data obtained in this study and existing literature. The causal relationships and detailed molecular pathways require further experimental validation.

Based on the temporal sequence of recovery observed in blood parameters and the cytokine profile, we propose a hypothetical tri-phasic model to conceptualize the potential integrated action of JOL (Figure 5):

**Phase I: inflammatory resolution and myeloid activation**. This initial phase is characterized by a rapid decline in systemic inflammation (marked reduction in TNF-α and IFN-γ), thereby removing a major barrier to hematopoietic recovery (39). Concurrently, myeloid hematopoiesis is preferentially activated, as evidenced by the early recovery of WBC, GR, and MID counts and supported by the coordinated upregulation of G-CSF and GM-CSF. The core objective of this phase is to dismantle the inflammatory milieu and launch robust myeloid regeneration to re-establish innate immune defense.

**Phase II: erythroid reconstitution and immune network repair**. This central repair phase focuses on correcting anemia and rebuilding adaptive immunity (40). A substantial surge in Erythropoietin (EPO) powerfully propels erythropoiesis, resulting in significant improvements in RBC, HGB, and HCT. The critical upregulation of Flt3-L indicates a foundational action on the HSPC pool, ensuring a sustainable source for multi-lineage reconstitution (41). The concomitant recovery of LYM counts and sustained suppression of IFN-γ signify the re-establishment of adaptive immune surveillance, transitioning the system into a comprehensive reparative state (42).

**Phase III: megakaryocytic maturation and systemic homeostasis**. The final consolidation phase involves the often-protracted recovery of the megakaryocytic-platelet axis. While complete PLT normalization may require more time, the observed increase in P-LCR and decrease in PDW unequivocally signal the bone marrow's release of fresh, functionally potent platelets and an overall trend toward platelet population homogeneity. This process is likely underpinned by subtly recovered TPO levels and the persistently ameliorated inflammatory environment (43), collaboratively fostering megakaryocyte maturation and the stabilization of platelet homeostasis. The delayed platelet recovery may reflect the longer maturation cycle of megakaryocytes or a differential sensitivity of megakaryocytic precursors to cyclophosphamide damage.

Notably, the therapeutic profile of JOL appears to differ from that of the conventional positive control, Ass-hide Gelatin (AE). AE, derived from donkey skin, is rich in collagen, peptides, and amino acids, and its hematinic effects are traditionally attributed to nutritional supplementation and broad-spectrum support for blood formation (44). In contrast, JOL comprises defined pectic oligosaccharides with low molecular weights, which may interact directly with pattern recognition receptors (e.g., toll-like receptors, C-type lectin receptors) on immune or stromal cells within the hematopoietic niche. This structural specificity could enable JOL to actively resolve inflammation, directly promote niche repair, and precisely modulate cytokine networks—a mechanism distinct from the nutrient-based support offered by AE.

In summary, JOL's therapeutic action appears to follow a coordinated, sequential process that begins with inflammation resolution, proceeds through core hematopoietic reconstruction, and culminates in systemic homeostasis.

A pivotal finding of this study is the significant suppression of the key inflammatory cytokines IFN-γ and TNF-α following JOL treatment. As these factors are known to directly impair HSPC function and disrupt the marrow niche, the efficacy of JOL can be partially ascribed to its potent anti-inflammatory properties. By mitigating inflammatory stress, JOL removes a critical impediment to the survival and proliferation of hematopoietic cells, thereby synergizing with upregulated growth factors to foster a conducive reparative milieu. The documented immunomodulatory capacity of jujube-derived oligosaccharides, as degraded products of plant polysaccharides (45, 46), offers a plausible mechanistic basis for this observed anti-inflammatory effect. This schematic model is proposed based on correlative data from this study and existing literature. The causal relationships, temporal boundaries between phases, and detailed molecular pathways require further experimental validation.

## 5 Conclusion and prospects

In conclusion, this study preliminarily confirms that Jujube Oligosaccharides (JOL) have a definitive therapeutic effect on cyclophosphamide-induced myelosuppressive anemia. The mechanism involves not a single pathway, but a synergistic, multi-target process encompassing: activation of multi-lineage hematopoiesis, repair of the hematopoietic microenvironment structure, positive regulation of hematopoietic growth factors, and suppression of inflammatory factors. This “multi-pronged” mode of action may explain its superior efficacy compared to the traditional drug Ass-hide Gelatin.

### 5.1 Limitations and future perspectives

A limitation of this study is that the specific molecular targets and the precise signaling pathways through which JOL acts remain unclear. Additionally, the therapeutic effects were evaluated at a single dose; thus, a dose-response relationship for JOL has not been established. Future research should focus on further isolating and identifying the active monomeric components within JOL. Utilizing *in vitro* hematopoietic progenitor cell cultures, gene knockout models, and dose-response assessments will allow for a precise dissection of its mechanism of action at the cellular and molecular levels, providing a solid theoretical basis for its development as a novel protective agent against radiation or chemotherapy-induced myelosuppression. Future studies employing *in vitro* cultures of bone marrow-derived hematopoietic progenitor cells, or coculture systems with stromal cells, would help elucidate the direct cellular targets of JOL.

## Data Availability

The raw data supporting the conclusions of this article will be made available by the authors, without undue reservation.
